# Emerging Exosomes and Exosomal MiRNAs in Spinal Cord Injury

**DOI:** 10.3389/fcell.2021.703989

**Published:** 2021-07-09

**Authors:** Jia Feng, Yifan Zhang, Zhihan Zhu, Chenyang Gu, Ahmed Waqas, Lukui Chen

**Affiliations:** ^1^Department of Neurosurgery, Neuroscience Center, Cancer Center, Integrated Hospital of Traditional Chinese Medicine, Southern Medical University, Guangzhou, China; ^2^School of Medicine, Southeast University, Nanjing, China

**Keywords:** exosome, spinal cord injury, miRNA, delivery vehicle, hydrogel

## Abstract

Acute spinal cord injury (SCI) is a serious traumatic event to the spinal cord with considerable morbidity and mortality. This injury leads to short- and long-term variations in the spinal cord, and can have a serious effect on the patient’s sensory, motor, or autonomic functions. Due to the complicated pathological process of SCI, there is currently no successful clinical treatment strategy. Exosomes, extracellular vesicles (EVs) with a double-layer membrane structure of 30–150 nm diameter, have recently been considered as critical mediators for communication between cells and tissues by transferring proteins, lipids, and nucleic acids. Further studies verified that exosomes participate in the pathophysiological process of several diseases, including cancer, neurodegenerative diseases, and cardiovascular diseases, and could have a significant impact in their treatment. As natural carriers of biologically active cargos, exosomes have emerged as pathological mediators of SCI. In this review article, we critically discuss the functions of exosomes as intracellular mediators and potential treatments in SCI and provide an outlook on future research.

## Introduction

**FIGURE 1:**
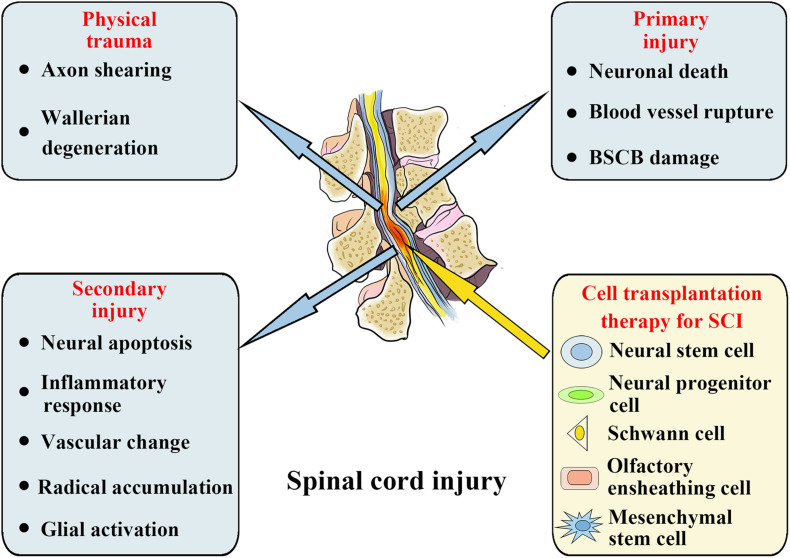
Spinal cord injury: Pathophysiological events and potential stem cell treatments.

Acute spinal cord injury (SCI) is a severe traumatic event to the spinal cord. On the basis of World Health Organization (WHO) estimates, there are annually approximately 250,000–500,000 new cases globally of SCI (Fehlings et al., 2017). When spinal trauma occurs, the SCI patient’s normal sensory, motor, or autonomic function will be significantly affected. On the basis of many initial primary injuries, progressive secondary injuries can worsen the clinical situation (Witiw and Fehlings, 2015). Primary injuries are mainly caused by misalignment and damage of the spine, leading to severe SCIs, including spinal cord contusion, compression, and transaction. When a primary injury occurs, neuronal death, blood vessel rupture, and blood spinal cord barrier (BSCB) damage occur immediately. Following the primary injury occurrence, a further secondary injury will result at the SCI site, causing further neurological damage and dysfunction. The secondary injury consists of neural apoptosis, inflammatory response, vascular change, radical accumulation, and glial activation, which will further extend the SCI (Figure 1; Silva et al., 2014; Tran et al., 2018). Therefore, potential treatment methods must also consider various acute-stage pathophysiological processes, including neural apoptosis, vascular changes, oxidative stress, and inflammatory responses. During the chronic phase, the main therapeutic goals include reversing demyelination, stimulating axon regeneration and sprouting, and inhibiting scar formation (Hilton and Bradke, 2017; Orr and Gensel, 2018; Sun et al., 2018; Ogata, 2019). However, because of the complicated progression of SCI, treating it presents a significant obstacle. Despite decades of study into the anatomy and physiological pathways of SCI, there are currently no appropriate therapeutic methods for its therapy.

Researchers have investigated the applications of neural cell transplantation in SCI over the past decade and have made significant progress. At present, many different types of strategies for cell transplantation are being analyzed in SCI treatment, including neural cell transplantation, such as neural progenitor and stem cells, and non-neural cell transplantation, such as olfactory ensheathing cells, Schwann cells, and mesenchymal stem cells (MSCs) (Figure 1; Cerqueira et al., 2018; Gomez et al., 2018; Ma et al., 2019; Sankavaram et al., 2019; Li L. et al., 2020). In addition, cell transplantation has been shown to be a promising therapeutic alternative for SCI in a number of early stage clinical trials, but feasibility and long-term protection have yet to be determined (Feron et al., 2005; Saberi et al., 2011; Curtis et al., 2018). Despite the extensive research on cell transplantation strategies for SCI management, the mechanism has still to be clarified how transplant cells provide an interaction with the host cells, either directly or indirectly, thereby promoting repair and mediating functional improvement. In addition, stem cell therapy is limited by tumorigenicity, low survival rate, and immune rejection, such that the clinical applications of stem cell cells are frequently restricted by a variety of factors (Liu et al., 2021). Additionally, glial scar formation prevents the integration, differentiation, and axon regeneration of transplanted stem cells in the diseased area, resulting in permanent functional defects. Despite this, there is increasing evidence that the beneficial results of this cell-based therapy are facilitated by exosomes discharged by donor cells, and the microRNAs (miRNAs) in these exosomes have a substantial impact on SCI management (Zhang Z.G. et al., 2019). For these reasons, ever more research groups have begun to explore the positive role of exosomes in SCI treatment and discover the potential mechanisms of exosome and exosomal miRNAs treatment.

## Cellular and Molecular Mechanistic Changes Following SCI

### Cellular Mechanistic Changes

Cell level changes after SCI mainly include infiltration of inflammatory cells, cell necrosis death and loss caused by various mechanisms, astrocyte activation and proliferation, apoptosis of oligodendrocytes and neurons, and the response of oligodendrocytes and their precursors (Ahuja et al., 2017).

#### Neuronal Apoptosis

Neuronal apoptosis after SCI may occur by cell surface signaling pathways involving “death receptors,” which are members of the tumor necrosis factor (TNF) receptor family, including TNF receptor 1, Fas, Fas ligand, p75, and DR3. These receptors can recruit and activate the caspase-8 and caspase-10 genes in the “dead area” of cells through them, thus inducing apoptosis. Following SCI, the Fas and p75 receptors are expressed on the surface of oligodendrocytes, astrocytes, and microglia in the spinal cord (Xu et al., 2018). Apoptosis occurs as early as a few hours after injury, and continues to occur in anterograde and retrograde regions, including brain regions, during the chronic phase of injury (Orr and Gensel, 2018).

#### Inflammatory Cell Response

Because of the protective effect of the blood–brain barrier (BBB), the central nervous system (CNS) is relatively immune amnesty. However, as a result of SCI, the BBB integrity is destroyed, leakage of blood vessels is increased, and immune cells can invade the CNS, inducing the occurrence of an inflammatory reaction, leading to the activation of local immune cells and the recruitment of immune cells (Kumar et al., 2017; Cash and Theus, 2020).

The inflammatory events after SCI are the cascade reactions of complement, cellular reaction, including local microglia activation and leukocyte macrophage infiltration, and the formation and proliferation of reactive astrocytes. The peak of microglia activation in the injured center occurs 3–7 days after injury, while, the peak of monocyte infiltration and macrophage activation occur within 7 days of injury. The binding of proteins, myelin fragments, and other cells in spinal cord tissue with CR3, FC, and toll-like receptors on neutrophils and macrophages can further trigger the release of inflammatory mediators. At the same time, the injured spinal cord also begins to express some inflammatory factors and related receptors (Ahuja et al., 2017).

Under normal conditions, activated T lymphocytes can cross the blood brain/spinal cord barrier to enter the CNS parenchyma. Thus, there are a small number of T lymphocytes scattered in the spinal cord. However, during the first week after SCI, the number of T cells in the injured center increases gradually and microglia activation and peripheral macrophage infiltration occur simultaneously. Although the number of T lymphocytes is less than other recruited cells (Orr and Gensel, 2018), it still plays an important role in the CNS immune system, which can produce a variety of cytokines and kill target cells.

The role of the inflammatory reaction in SCI is very complex. With the progress of time after the SCI, cells that come from the immune and nervous systems have different reactions and interactions at different stages. Like other parts of the body, the inflammatory reaction after SCI is an inevitable reaction, and it is a trauma and an indispensable process of tissue repair. The inflammatory reaction has different effects at different stages in SCI. The early inflammatory reaction can further exacerbate the tissue injury, whereas in the later stage, a variety of growth factors and nutritional factors secreted by inflammatory cells are essential for tissue repair.

#### Astrocyte Reaction and Proliferation

The first phenomenon after SCI is cell death. In the initial stage of SCI, this phenomenon is limited to the damaged area. However, the damage gradually expands during the following days, and a wide range of astrocyte reactions are initiated. An early event that affects the astrocyte response is the invasion of spinal membrane cells into the damaged area, forming a new glial boundary membrane between these cells and the surviving astrocytes. The astrocytes around this area become hypertrophic and divide. The final result is the formation of glial scars, which are mainly composed of astrocytes (Bradbury and Burnside, 2019). Astrocytes also undergo many other biochemical changes, including the production of nutrients, cytokines, proteases, protease inhibitors, matrix, and other molecules.

### Molecular Mechanistic Changes

The changes in the level of different molecules after SCI are complex. A variety of cell components contained in injured tissues can produce a variety of cytokines and chemical factors, including not only harmful factors that can cause tissue damage and prevent regeneration but also beneficial factors that can maintain cell survival and rescue cells from death. These factors will change at different stages of the SCI, and they interact with each other, which makes it very difficult to understand their different changes and effects after injury.

#### Molecular Regulation of Self-Repair After SCI

Following SCI, myelin fragments and extracellular matrix in the injured area can secrete a series of inhibitors of nerve regeneration to inhibit the self-repair of spinal cord, this limiting the repair effect. Among them, myelin-associated glycoprotein can inhibit neurogenesis and induce axon growth bundle retraction (Jurynczyk et al., 2019), oligodendrocyte-associated myelin glycoprotein can inhibit neurogenesis and stump regeneration (Reindl and Waters, 2019), and CSPG can limit axon plasticity and remyelination (Sami et al., 2020).

During the chronic repair period after SCI, glial scar formed by astrocytes and other cells in the damaged area will secrete a series of inhibitory proteins and cytokines, including NG2 glycoprotein, to inhibit axon regeneration (Hackett and Lee, 2016; Tai et al., 2021). Therefore, the specific inhibition of these cytokines and growth factors and the transmission of their inhibitors are particularly important.

#### Signal Transduction Pathways of Self-Repair After SCI

Following SCI, many signal transduction pathways with different functions are activated and involved in the related damage and repair mechanisms, which form a complex signal interaction network. As one of the important pathways regulating cell proliferation and apoptosis, the mitogen-activated protein kinase (MAPK) signal transduction pathway can be activated by inflammatory cytokines and oxidative stress products released from SCI-damaged areas, and participate in the proliferation and apoptosis of injured local cells (Liu et al., 2017). The Janus kinase/signal transducer and activator of transcription (JAK/STAT) signal transduction pathway is an important pathway to transfer signals from the cell surface to the nucleus, which can participate in the regulation of the normal cell cycle and various immune inflammatory reactions (Dai et al., 2018a, b). As a complex serine threonine protein kinase protein, the mammalian target protein of rapamycin (mTOR) signal transduction pathway can participate in the regulation of cell growth and differentiation, particularly apoptosis (one of the important pathophysiological reactions following SCI) (Saxton and Sabatini, 2017; Zhou et al., 2018). Although in the development process of SCI, the compositions and functions of the MAPK, JAK/STAT, and mTOR signal transduction pathways have not been thoroughly researched, these three signal transduction pathways have been confirmed to be involved in the injury and repair of SCI, thus providing us with new ideas for exploring further treatment options for SCI.

## Exosomes and Exosomal Mirnas

### Exosome Biogenesis

**FIGURE 2:**
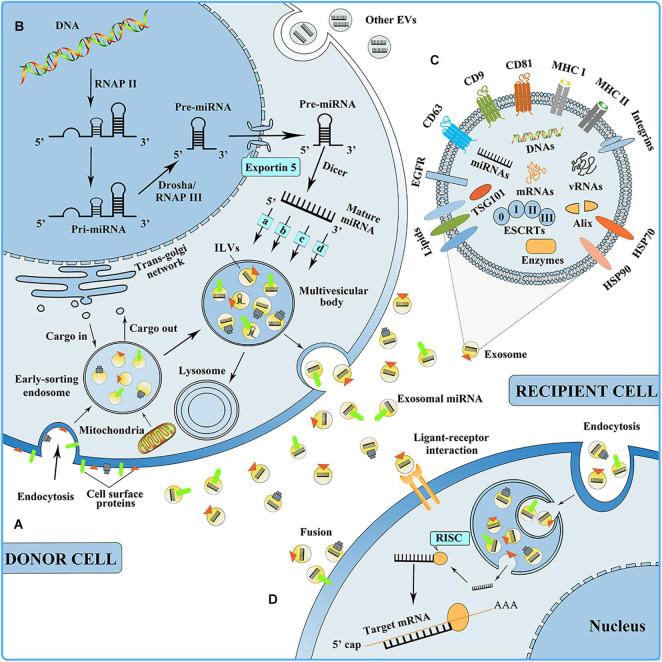
**(A)** Exosome biogenesis. EVs, extracellular vesicles. **(B)** miRNA biogenesis, sorting into exosome, and mediating intercellular communication. RNAP II, RNA polymerase II; RNAP III, RNA polymerase III; (a) nSMase2-dependent pathway; (b) the miRISC-related pathway; (c) 3′miRNA sequence-dependent pathway; (d) miRNA motif and sumoylated hnRNPs-dependent pathway. RISC, RNA-induced silencing complex. **(C)** Exosome composition. ESCRTs, endosomal-sorting complex essential for the transport, including ESCRT 0, ESCRT I, ESCRT II, and ESCRT III; EGFR, epidermal growth factor receptor; HSP, heat shock protein. **(D)** Exosomes can be taken up by the recipient cell in three ways: Endocytosis, ligand-receptor interaction, and fusion.

Exosomes are small extracellular particles that range in size from 30 to 150 nm in diameter. They have an endosomal origin, have the same topology as a cell, and are generated by nearly all of the body’s cells (Kalluri and Lebleu, 2020). Exosome biogenesis is subject to strict biological regulation. The cellular membrane invagination is the first phase of exosome biogenesis, with some cell-surface proteins and soluble proteins, and with the participation of the trans-Golgi network and the endoplasmic reticulum, thereby forming an early sorting endosome (ESE) (Huotari and Helenius, 2011; Hessvik and Llorente, 2018). Late-sorting endosomes can develop from ESEs with the participation of the endosomal-sorting complex necessary for transport (ESCRT) proteins, the inward germination of the membrane encapsulates biomolecules, and intraluminal vesicles (ILVs, future exosomes) are generated within the multivesicular bodies (MVBs), which are also called multivesicular endosomes (Simons and Raposo, 2009; Zhu et al., 2019; Vietri et al., 2020). MVBs simultaneously follow one of two paths, one is to combine with lysosomes or autophagosomes to be degraded, and the other is fusion to the plasma membrane to release ILVs as exosomes (Figure 2; van Niel et al., 2018). When exosomes are discharged, they can reach adjacent tissues and organs or circulate inside body fluids (for example, blood, and cerebrospinal fluid) to reach distant tissues and organs (including the lung, liver, spleen, and gastrointestinal tract) and be taken up by the recipient cells in three ways: Endocytosis, direct fusion with the plasma membrane, and ligand-receptor interaction (Figure 2; van Dongen et al., 2016; Riau et al., 2019). Moreover, it is speculated that after their uptake by the recipient cells, exosomes can undergo back-fusion at MVBs to release their functional cargo, including RNAs, DNAs, and proteins, and participate in the body’s physiological processes.

### Exosome Compositions

With the participation of ESCRT proteins, various biomolecules are encapsulated during the formation of ILVs in MVBs, and these biomolecules constitute the composition of exosomes. Electron microscopy and proteomic techniques are used to determine the exosome compositions and find various biomolecules, such as proteins (including receptors, adhesion molecules, tetraspanin proteins, and enzymes), lipids (including cholesterol, sphingolipids, and ceramide), and nucleic acids (including DNAs, mRNAs, and miRNAs) within and on the exosome surface (Figure 2; Jeppesen et al., 2019; Pegtel and Gould, 2019). Among the exosomal proteins, there are both ubiquitous and cell-specific proteins, including cytoskeletal proteins, annexins, and Rab proteins. Exosomes also contain heat-shock proteins (including HSP70 and HSP90); these are implicated in antigen presentation (Thery et al., 2002). Additionally, abundantly expressed tetraspanin proteins are found on exosomes from most cell types, the representative of which are CD9, CD63, and CD81, and their enrichment in exosomes makes them recognized exosome marker proteins (Tian Y. et al., 2020). The lipid content in exosomes is relatively conservative, contributing to exosome biogenesis and playing a role in exosome form maintenance (Mashouri et al., 2019). Exosome RNAs are released to function between cells and tissues. Exosomal miRNAs are the most critical functional substances among the exosomal nucleic acids and silence post-transcriptional mRNA expression that is involved in various biological mechanisms, for example, cell proliferation and differentiation, immunomodulation, and angiogenesis (Gebert and Macrae, 2019; Treiber et al., 2019). However, the functions of exosomal DNAs and the mechanism by which they are encapsulated into exosomes remain to be fully elucidated.

### Mechanisms of miRNA Biogenesis and Sorting Into Exosomes

miRNAs are single-stranded short non-coding RNAs (ncRNAs) and can promote mRNA degradation by directly binding recognition motifs in the 3′-untranslated region (UTR) of the target mRNA, thereby inhibiting target gene expression (Treiber et al., 2019). miRNA biogenesis is strictly regulated at both the transcriptional and post-transcriptional levels. Its biogenesis occurs first in the nucleus, and the miRNA genes are subsequently transcribed by RNA polymerase (RNase) II to synthesize primary transcripts, which contain imperfect and self-complementary foldback regions. The pri-miRNA is sliced by the “Drosha microprocessor” complex and RNase III to establish a hairpin precursor miRNA (pre-miRNA) of approximately 60–70 nt. The pre-miRNA is transported and released within the cytoplasm by the nuclear complex composed of Xpo5 and RanGTP and further processed by the RNase III enzymes Dicer and transactivation-responsive RNA-binding protein (TRBP) to form mature miRNA. At the same time, mature miRNAs are packaged into exosomes through four different modes, including the (a) nSMase2-dependent pathway, (b) miRISC-relatedpathway, (c) 3′miRNA sequence-dependent pathway, and (d) miRNA motif and sumoylated hnRNPs-dependent pathway. Finally, MVBs merge together with the plasma membrane, and mature miRNAs are discharged into the extracellular region within the exosomes, where they participate in a variety of biological processes, including cell proliferation and differentiation, immunomodulation, and angiogenesis (Figure 2).

### Strategies for Exosomal Crossing of the BBB

The BBB is a highly dynamic physical barrier that separates the brain from the peripheral circulation. It controls the inflow and efflux of chemicals from the brain to maintain CNS homeostasis and the stable local ionic environment required for neuronal activity (Armulik et al., 2010; Daneman et al., 2010). Exosomes perform crucial functions in the communication process across the CNS, having effects on nearby and distant cells (Zhang and Yang, 2018). Material may be transported across the BBB in two ways: transcellularly through brain microvascular endothelial cells (BMECs) or paracellularly through synapses between BMECs (Chen et al., 2016).

Many types of research have been performed since the discovery that exosomes may penetrate the BBB and retain their activity. In mice, one study achieved efficient siRNA delivery to the brain by systemic exosome injection (Alvarez-Erviti et al., 2011). The authors modified dendritic cells to express the exosomal membrane protein lysosome-associated membrane protein 2 (Lamp2b). By linking Lamp2b to a CNS-specific rabies virus glycoprotein (RVG) peptide, the exosomes were specifically directed to the brain.

Exosomes generated from human erythrocytes were transported by adsorptive transcytosis in an *in vitro* BBB model (Matsumoto et al., 2017). Another study revealed that exosomes could cross the BBB under healthy and stroke-like conditions through transcellular BMEC endocytosis, indicating that exosomes maintain their capacity to cross under stressful conditions (Chen et al., 2016).

In addition to crossing the BBB, current research has shown a function for exosomes in increasing the permeability of BBB vascular barriers. For example, exosomes generated by breast cancer cells are the only ones that produce miR-105, a small RNA that directly targets the tight junction protein zonula occludens 1 (ZO-1). Translocation of this miR-105 exosome disrupts tight junctions and BBB integrity (Zhou et al., 2014). Additionally, it has been shown that claudin-5 (Cldn5) is contained in encapsulated exosomes, a type of tight junction protein found in the BBB. Cldn5 deficiency resulted in a relaxation of the BBB in mice, indicating that exosomes containing Cldn5 may contribute to BBB integrity (Paul et al., 2016). This study provides new information on this process and highlights the need for greater knowledge regarding exosomes as a mode of BBB penetration.

### Reasons for Exosomes as Transporter or Messenger

Research on exosomes has surged recently because of their exceptional features. Transport within exosomes enables concurrent intercellular communication by transmitting multiple signals at the same time (Armstrong et al., 2017). Additionally, cargos contained within exosomes are shielded from hydrolysis by enzymes and from other processes by their lipid bilayer, which confers stability and safety (Ha et al., 2016). Because of their tiny size and animal origin, exosomes can resist phagocytosis, merge with the cell membrane, and escape engulfment by lysosomes (van der Meel et al., 2014). Exosomes can potentially be employed to transport RNA (siRNA) or pharmaceutically active compounds (Li et al., 2017). They can display excellent stability in the bloodstream, allowing them to travel vast distances throughout the body under normal or pathophysiological conditions. Additionally, exosomes include a hydrophilic center, which makes them ideal for encapsulating water-soluble medicines (Jiang and Gao, 2017).

Nanoscale medication delivery devices are becoming ever more popular. To increase the therapeutic efficacy of chemical and biomolecular drugs, many nano-based medication formulations have been developed. Exosomes have gained considerable attention because of the discovery that they might operate as intercellular communication agents that deliver their contents to recipient cells (Luan et al., 2017).

The optimal medication delivery system should be capable of delivering treatments to specific locations. Exosomes can transport indigenous biological cargo between cells, including short RNAs, mRNAs, and proteins. They demonstrate several advantages in terms of biocompatibility and decreased clearance rates because of their natural composition (Goh et al., 2017). For these reasons, exosomes are thus a safe and durable endogenous nanocarrier and one of the most effective drug delivery methods, with an increasing number of applications.

### Strategies for Exosomes as Delivery Vehicles of Exogenous Cargos

To successfully deliver drugs and genes, it is essential to determine the optimal type of carrier to be utilized (Wahlgren et al., 2012). The following essential qualities must characterize a vesicle to facilitate optimal medication delivery: (i) is capable of encapsulating a sufficient amount of drug to provide a therapeutic effect, (ii) has prolonged constancy of size, shape, and therapeutic agent bioactivity throughout the blood circulation, and (iii) can bypass macrophages and is unharmful, immune-suppressive, and non-immunogenic (Arslan et al., 2013).

Exosomal vehicles combine the benefits of cell-based drug delivery with nanotechnology to produce effective drug transport (Escudier et al., 2005). Currently, the most significant scientific challenge is the question of how to successfully synthesize exosome-based drug products. Numerous loading techniques have been applied to insert drugs in this delivery system, mainly incubation, electroporation, and sonication (Munagala et al., 2016). In this section, we describe various approaches for combining exosomes and medicines.

(1) Incubation:

Incubation is probably the simplest method for combining medicines with exosomes (Kim et al., 2016). One study reported that curcumin-loaded exosomes might treat LPS-induced septic shock (Sun et al., 2010). Additionally, by culturing paclitaxel (PTX) with MSCs, we were able to generate PTX-loaded and MSC-derived exosomes with a substantial antitumor impact (Pascucci et al., 2014).

(2) Electroporation:

The use of rapid, high-voltage pulses to penetrate the exosomal cell membrane layer is known as electroporation (Gehl, 2003). Electroporating a solution of medicines and exosomes at 1,000 kV for 5 ms correctly loads the medicine into the exosomes (Kim et al., 2013). *In vivo* studies have shown that exosomes loaded with doxycycline by electroporation have targeted tumor tissues, resulting in tumor suppression without any risk of damage to normal cells (Tian et al., 2014).

(3) Sonication:

Sonication is a physical method that involves applying an additional mechanical shear strain to the exosomal membrane to undermine its integrity, allowing medicines, proteins, and nanoparticles to be loaded into the exosome ([Bibr B44][Bibr B73]). For example, PTX-loaded exosomes can be generated by sonication, which has a larger loading capacity than typical incubation methods (Haney et al., 2020). Additionally, sonication might be used to load catalase and gold nanoparticles into exosomes (Sancho-Albero et al., 2019). Therefore, sonication is a valuable technique for loading drugs into exosomes, but the membrane damage caused by sonication remains a significant barrier to its wide-scale use.

## Exosomes: Applications in SCI

**FIGURE 3:**
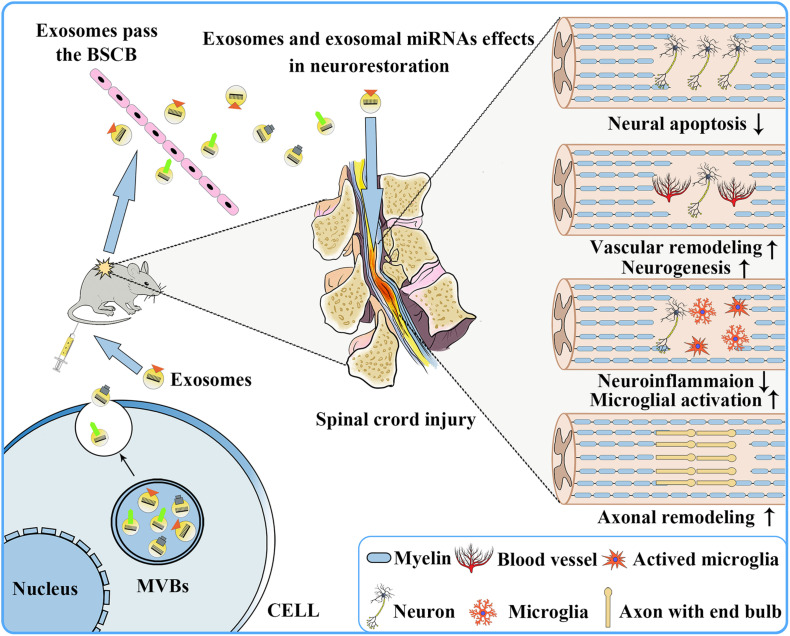
Repair of the nervous system following SCI after exosome transplantation. Exosomes secreted by donor cells can freely pass through the blood–brain barrier and play a role in promoting nervous system repair, including reducing neuronal apoptosis, promoting vascular remodeling and neurogenesis, reducing neuroinflammation, and promoting microglia activation and axonal remodeling.

When the spinal cord is physically injured, a large number of blood vessels rupture, resulting in bleeding, ischemia, and inflammation, and subsequently a large number of neuronal cells undergo death and apoptosis, microglia activate, and axons are lost within the damaged zone (Wong et al., 2020). Therefore, in the early stage of SCI, if one can effectively reduce neuronal cells apoptosis, promote vascular remodeling, stimulate neuronal regeneration, promote axon regeneration, regulate microglial activation, and inhibit inflammation, it is possible to effectively slow down the process of SCI and improve the prognosis of SCI patients. Because of the presence of various therapeutic growth factors and miRNAs in exosomes (Tomasoni et al., 2013; Anderson et al., 2016; Chen et al., 2021), in recent years, many studies have reported that exosomes have shown therapeutic effects on CNS diseases, including SCI (Table 1 and Figure 3).

**TABLE 1:** The roles of exosomal miRNAs in SCI.

miRNA s	Donor cells	Pathway	Function	Exosomes administration method	References
miR-133b	MSCs	ERK1/2, STAT3 and CREB	Attenuating neuronal apoptosis	Tail vein injection	Li D. et al., 2018
miR-21	MSCs	PTEN/PDCD4		Intravenous injection	Kang et al., 2019
miR-21, miR-9b	PC12 cells and MSCs	Targeting PTEN		Intravenous injection	Xu et al., 2019
miR-21, miR-9b	MSCs			Intravenous injection	Wang Z. et al., 2020
miR-126	MSCs	SPRED1, PIK3R2		Tail vein injection	Huang et al., 2020
miR-29b	BMSCs	N/A		Intravenous injection	Yu et al., 2019
miR-29b	HNESCs	PTEN, caspase-3		Intravenous injection	Kang et al., 2020
miR-199a-3p/145-5p	hUC-MSCs	NGF/TrkA	Promoting angiogenesis, neurogenesis and axonal remodeling	Tail vein injection	Wang et al., 2021
miR-92a	K562 cells	Targeting integrin α5		N/A	Umezu et al., 2013
miR-216a-5p	MSCs	TLR4/NF-κB/PI3K/AKT	Regulating neuroinflammatory response and microglia activation	Tail vein injection	Liu et al., 2020
miR-124-3p	Neurons	PI3K/Akt/NF-κB		Tail vein injection	Jiang et al., 2020
miR-155	M1-BMDMs	SOCS6/NF-κB		Tail vein injection	Ge et al., 2021

### Exosomes and Exosomal miRNAs Improve the Recovery From SCI by Attenuating Neuronal Apoptosis

#### Exosomes Improve SCI Recovery

Apoptosis is a programmed, ATP-driven process of cell death, and under the intervention of certain factors, such as exosomes, cell apoptosis can be attenuated (Grossman et al., 2000). It has been reported that neuronal apoptosis only occurs in the early phases of SCI (Singh et al., 2019). Therefore, research has confirmed that when exosomes are used for treatment in the early stage after SCI occurs, they can successfully attenuate neuronal cell apoptosis. For example, in two preclinical experiments in which SCI occurred in SCI mouse models, MSC exosomes (MSC-exosomes) after systemic administration could increase the expression level of the anti-apoptotic protein B-cell lymphoma 2 (Bcl2), whereas the expression level of pro-apoptotic protein Bcl-2-associated X protein (BaX) was significantly reduced, thereby promoting an improvement of the function of SCI rats (Huang et al., 2017; Yuan et al., 2019). Another preclinical experiment confirmed the anti-apoptotic effect of MSC-exosomes and demonstrated that they could effectively activate the Wnt/β-catenin signaling pathway to have an anti-apoptotic effect (Li et al., 2019). In addition, a study reported that MSC-exosomes could improve the expression of autophagy-related proteins, including LC3IIB and Beclin-1, and induce autophagosome formation. Simultaneously, the expression level of pro-apoptotic protein cleaved caspase-3 was notably reduced, whereas that of anti-apoptotic protein Bcl-2 was up-regulated, thereby reducing neuronal apoptosis by nearly 70% (Liu et al., 2019; Gu et al., 2020). These studies indicate that BMSC-exosomes might be a potential management approach for SCI.

#### Exosomal miRNAs Improve SCI Recovery

As is well known, exosomal miRNAs are important components of the exosome functional substances, and are thought to play an important role in the processes of reduced neuronal apoptosis induced by exosomes. For example, when miR-133b-encapsulated exosomes were injected into SCI rats, STAT3, ERK1/2, and CREB were activated, damaged neurons were protected, and the restoration of hindlimb locomotor function of SCI rats was improved (Li D. et al., 2018). miR-21-rich and miR-19b-rich exosomes can enhance neuronal viability and inhibit neuronal death by inhibiting PTEN/PDCD4 expression (Kang et al., 2019; Xu et al., 2019; Wang Z. et al., 2020). Additionally, exosomes originating from miR-126-modified MSCs have also been seen to reduce neuronal apoptosis and facilitate functional regeneration after SCI (Huang et al., 2020). The exosomes secreted by miRNA-29b-rich MSCs and human neuroepithelial stem cells can have a therapeutic effect on SCI by down-regulating PTEN/caspase-3 expression and subsequently inhibiting neuronal cell apoptosis (Yu et al., 2019; Kang et al., 2020). The above research results indicate that when SCI occurs, exosomes can enhance neuronal cell activity and attenuate its apoptosis at an early stage through their miRNA transport, thereby promoting functional recovery, thus proving that exosome-mediated miRNA transfer represents a new method for treating SCI.

### Exosomes and Exosomal miRNAs Improve SCI Recovery by Promoting Angiogenesis, Neurogenesis, and Axonal Remodeling

#### Exosomes Improve SCI Recovery

Direct vascular injury after SCI can cause bleeding and inflammation, thereby exacerbating SCI. Previous studies have confirmed that new axons will grow along blood vessels (Xue et al., 2018), such that after SCI, abnormal angiogenesis will reduce endogenous neural tissue repair and tissue regeneration. Therefore, promoting angiogenesis after SCI can promote neurogenesis and axonal remodeling, thereby improving neurological function. The pro-angiogenesis role of exosomes makes them a new potential target for SCI treatment.

When SCI occurs, the injured spinal column is hypoxic. As a necessary component of the blood vessel wall, vascular endothelial cells elevate the uptake of exosomes produced by hypoxia-treated MSCs. The ingested exosomes can activate the protein kinase A (PKA) signaling path and promote VEGF expression and consequently angiogenesis (Rauch et al., 2009). Human urine stem cell-derived exosomes can pass through the BSCB and transport ANGPTL3 protein to the SCI area, stimulating angiogenesis through the PI3K/AKT signaling pathway, thereby enabling SCI recovery (Cao et al., 2021). MSC-exosomes packed with phosphatase and tensin homologous small interfering RNA (ExoPTEN) can significantly enhance the angiogenesis and axon regeneration in the damaged spinal cord by reducing PTEN expression in the damaged spinal cord area while reducing microglia and astrocyte proliferation, thereby significantly improving the functional recovery of SCI rats (Kim et al., 2018). Exosomes generated by MSCs have also been confirmed to decrease the permeability of the BSCB, enhance its integrity, and promote axon regeneration by down-regulating the NF-κB p65 signaling pathway in pericytes (Lu et al., 2019). When SCI occurs, if exosomes can be given early intervention to promote angiogenesis and axonal remodeling, it may have a positive effect on the functional recovery of SCI patients. This requires more and more in-depth experiments to verify.

#### Exosomal miRNAs Improve SCI Recovery

To date, several studies have shown that miRNAs transported by exosomes from various cell origins have a significant protective role in SCI. For example, a preclinical study confirmed that miRNAs with higher expression levels in exosomes, including miR-199a-3p/145-5p, can target the Cblb and Cbl genes to affect TrkA ubiquitination and activate the NGF/TrkA pathway to encourage axon development and motor control recovery in rats with SCI (Wang et al., 2021). Another study demonstrated that miR-92a from K562 cell exosomes significantly promotes endothelial cell tube formation and migration (Umezu et al., 2013). Many scientists have investigated the application of plasma in the recovery of soft tissues in recent years. Studies have demonstrated that exosomes induce angiogenesis by transferring biologically active molecules, including miRNAs. One study isolated human umbilical cord blood exosomes (UCB-exos) from plasma and subcutaneously injected them into wound sites of mice. The results showed that UCB-exos reduced scar width and enhanced angiogenesis. Further studies showed that the most highly expressed miRNA in UCB-exos, miR-21-3p, can be transferred into fibroblasts and endothelial cells, whereby it can inhibit PTEN and sprouting homolog 1 to induce the activation of the PI3K/Akt and ERK1/2 pathways (Hu et al., 2018). That is, UCB-exos can play a positive role in mediating angiogenesis and promoting fibroblast proliferation, collagen synthesis, and migration by miR-21-3p.

### Exosomes and Exosomal miRNAs Improve SCI Recovery by Altering Neuroinflammatory Potential, Regulating Microglia Activation, and Forming a Neuroprotective Scar

#### Exosomes Improve SCI Recovery

SCI activates inflammatory responses. This neuroinflammation is a form of innate immune response caused by microglia and astrocytes (Pinchi et al., 2019). It is important to understand the neuroinflammatory response in the CNS. Microglia is a neural cell type of the CNS, and is essential for maintaining healthy brain homeostasis and neuropathology (Bellver-Landete et al., 2019). Following SCI, hypertrophy and neurite expansion of astrocytes occur around the lesion. These reactive astrocytes migrate to the lesion center and promote tissue repair. Subsequently, reactive astrocytes form glial scars, produce axon growth inhibitors, and prevent CNS axon regeneration. In addition, astrocyte exosomes in the blood can stimulate organs to produce cytokine and chemokine gene responses to CNS inflammation.

One study demonstrated the alleviation of SCI-induced neuropathic pain by 17β-estradiol, in that 17β-estradiol could inhibit microglia and astrocyte activation and the resultant inflammation (Lee et al., 2018). Exosomes could freely cross the BBB and participate in the physio-pathological processes of many neuroinflammatory diseases. The RARβ agonist was found to be able to inactivate phosphatase and tensin homologs in neurons, and to modulate axonal regeneration via PTEN phosphorylation by neuron–glia exosome transfer to reduce scar formation. This molecule might be a potential therapeutic goal for SCIs (Goncalves et al., 2015). Membrane-associated myelin-related inhibitors (MAIs) were one of the inhibitor groups of axon growth in the nervous system. Nogo-A, the most important MAI, was produced as an exosome protein after injury to act as an effective inhibitor of axonal regeneration (Sekine et al., 2020). *Clostridium botulinum* C3 exoenzyme (C3bot) can inhibited the Rho family, and it had been extensively used in SCI management (Fehlings et al., 2018). The intermediate filament protein vimentin can acted as a surface interaction partner of C3bot. It had been shown that exosomes secreted by reactive astrocytes were the source for extracellular vimentin. This mode provided a new mechanism for the neuroprotective effects of C3bot after SCI (Adolf et al., 2019). Exosomes produced from human umbilical cord MSCs (MExos) have been shown to facilitate neural stem cell (NSC) *in vitro* and *in vivo* migration. Because of this characteristic, MExos can be used as an effective drug carrier. Researchers utilized MExos to deliver PTX via a bio-specific peptide to promote nerve regeneration and reduce scar tissue deposition, which showed excellent performance in the re-establishment of motor skills after complete SCI in rats, such that this method can repair SCI in one step (Zhang et al., 2021).

#### Exosomal miRNAs Improve SCI Recovery

During SCI management, we should emphasize preventing M1 microglia and A1 astrocyte activation, as well as a high level of neuroinflammation. The imbalance of some miRNAs can lead to excessive microglia activation, neuroinflammation, and abnormal macrophage polarization. Much research had been performed exploring the significant therapeutic effect when strengthening the biological activity of exosomes through regulating miRNAs. miR-216a-5p was enriched in MSC-derived exosomes under hypoxia and modulated microglial polarization via the TLR4/NF-κB/PI3K/AKT pathway (Liu et al., 2020). This also suggested that hypoxic preconditioning might be an alternative treatment for SCI. miR-124-3p was the most abundant miRNA in neuron-derived exosomes. It exerted its inhibitory actions by inhibiting the activity of myosin heavy chain 9 (MYH9), then suppressed the stimulation of M1 microglia and A1 astrocytes. The miR-124-3p/MYH9 axis was found to aid functional rehabilitation following a SCI in mice by controlling the PI3K/Akt/NF-κB signaling (Jiang et al., 2020). Bioinformatics assessment indicated that miR-155 was enriched in M1-polarized microglia. *In vitro* study confirmed that miR-155 can be delivered by exosomes, and it inhibited SOCS6 expression and NF-κB signaling activation by suppressing SOCS6-induced p65 degradation. These studies demonstrated that the miR-155/SOCS6/p65 axis can upregulate the ROS level in the microvascular endothelial cell line (bEnd.3 cells) and lead to significant mitochondrial dysfunction. This research revealed a new method for maintaining BSCB integrity after SCI (Ge et al., 2021). The pro-inflammatory cytokines TNFα and interleukin (IL)-1β could upregulate some miRs (miR-145-5p, miR-24-3p, miR-214-3p, miR-206, and miR-34c-5p) and downregulate other miRs (miR-451a, miR-29b-3p, and miR-21-5p). Following SCI, the IL-1β and IL-1α levels were increased and the IL-6 level was decreased in astrocytes; this alteration may trigger some changes of miRs in exosomes (Chaudhuri et al., 2018). These results point to a new pathway for the use of neuron-derived exosomes as well as a possible goal for SCI therapy.

### Exosomes as a Diagnostic Marker of SCI

The evaluation of spinal injury is important for high-quality care. Exosomes can be obtained from the plasma, which can be retrieved reasonably easily when required. Exosomes are loaded with biologically active components, which provide considerable information for diagnosis and treatment (Jalalian et al., 2019). In addition, exosomes in biological fluids alter with the progression or occurrence of a disease, such that on their analysis, exosomes can undergo gradual modifications in their response to physiological and pathological processes *in vivo*.

We have already indicated that miRNAs are engaged in the process of SCI through a variety of mechanisms. Exosomal miRNAs have several advantages compared with the “free circulation” miRNAs (low concentration and poor stability) in bodily fluids (Manier et al., 2017; Sohel, 2020). Previous studies have proved that SCI leads to changes of miRNA expression. Several studies found highly enriched exosomal miRNAs after SCI, and these serum exosomal miRNAs are of considerable importance in the evaluation and prognosis of SCI. For example, in two independent preclinical studies, next-generation sequencing technology was used to analyze the differences in serum exosomal miRNA profiles of sham and acute SCI rats, confirming that miR-152-3p, miR-130a-3p, miR-597, and miR-1056 expression were significantly increased in exosomes in the acute SCI group, whereas miR-125b-5p, miR-47, and miR-99b-3p expression were significantly decreased (Ding et al., 2019, 2020), indicating that they may become specific and easy-to-detect biomarkers for acute SCI. Many further miRNAs are not considered here. In summary, although a single miRNA as a biomarker of SCI may lack specificity, it would be meaningful to combine multiple important exosomal miRNAs as informative biomarkers to form microarrays to guide treatment and aid in prognosis.

## Perspectives

### Exosomes as Drug Delivery Vehicles

Successful delivery of therapeutic agents to the target cells and tissues is restricted by numerous factors, including the instability of therapeutic agents *in vivo*, seclusion from target tissues, the activities of the BBB and BSCB, and the drug efflux system. By interfering with the arrival of the vast majority of aerotherapeutics and small molecules to the brain, the BBB and BSCB are among the most significant obstacles for treating CNS disorders. However, several specific properties of exosomes offer great potential for their use as drug delivery vehicles. For example, exosomes can transport biomolecules, pass through the BBB and BSCB, and reach distant organs, including the brain and spinal cord, without significant degradation. Therefore, researchers have engineered exosomes to make them therapeutic drug delivery vehicles.

#### Exosomes as Drug Delivery Vehicles for ncRNAs

ncRNAs are a form of RNA that cannot be translated into protein, comprising small ncRNAs (such as miRNAs) and long ncRNAs (lncRNAs) (Deniz et al., 2019), and play a vital regulatory role in maintaining cell activity.

For small ncRNAs, such as miRNAs, mature miRNAs can be delivered to recipient cells after being sorted in exosomes, thereby regulating multiple key homeostatic processes by regulating gene expression. Consequently, they can have a significant influence on the protein network and RNA production of the recipient cells. miRNAs have been found to play essential roles in all phases of neurodevelopment, neuroplasticity, and neurological disease progression of nervous system injuries, including stroke and SCI (Branscome et al., 2020). For example, in the pathophysiology of stroke, the miR-21-5p and miR-30a-5p levels can reflect different stages of stroke (Wang et al., 2018), miR-17-92 can play a therapeutic role (Xin et al., 2017), and miR-128 can play an early diagnosis role in the occurrence of stroke (Li S. et al., 2020). Additionally, miR-219 can be combined with miR-338 to promote remyelination and the recovery of the CNS (Wang et al., 2017). At this time, we can make a bold assumption that exosomal miRNAs might have a possible function in different stages and diagnosis of SCI, and when up-regulating the contents of related miRNAs in exosomes, this may have significant therapeutic value for SCI.

For lncRNAs, it has been demonstrated that lncRNAs can participate in many physio-pathological procedures, including the immune response, inflammatory response, and cell differentiation and proliferation, by regulating the stability and nuclear retention of their target genes (Lee and Nam, 2017). LncRNAs have been proved to play an essential role in the occurrence, development, and treatment of numerous types of cancers (Li Z. et al., 2020). The study of lncRNAs is becoming more in-depth. Abundant lncRNA expression in the CNS has been described to be closely related to CNS development and function, involving homeostasis, stress responses, and plasticity (Qureshi et al., 2010), and can play critical roles in numerous neurological injuries. For example, in a preclinical experiment, it was reported that the lncRNA ZNF667-AS1 could restrict the inflammatory response and promote the functional recovery of SCI rats by blocking the JAK-STAT pathway (Li J.W. et al., 2018). Additionally, clinical results indicated that SCI patients’ serum lncRNA-tSix levels are slightly greater than the control groups, which means that lncRNA-tSix can potentially be a biomarker for SCI diagnosis (Salah et al., 2020). As a result, exosomes that overexpress various lncRNAs may provide for their possible development as novel treatments for SCI and might perform a vital role in the future. In addition, some lncRNAs that are differentially expressed in exosomes could be used as biomarkers for SCI and provide the possibility of early SCI diagnosis.

#### Exosomes as Drug Delivery Vehicles for Cytokines

Cytokines and chemokines have been confirmed to be involved in the initiation, regulation, and spread of immune and inflammatory responses. Many previous articles have verified that cytokines and chemokines play an essential role in the pathophysiology of various neurological disorders, for example, Alzheimer’s disease, multiple sclerosis (Donninelli et al., 2020), stroke (Georgakis et al., 2019), and multiple sclerosis (Papadimitriou et al., 2018). Cytokines and chemokines such as IFN-γ, NGF, and BDNF are synthesized by glial cells and neurons in the nervous system and play essential roles. Ever more studies have focused on cytokines and chemokines in exosomes in neurological diseases, and breakthroughs have been achieved. For example, our research group’s previous preclinical study showed that human neural stem cell exosomes (IFN-γ-hNSC-Exo), induced by the pro-inflammatory factor IFN-γ, have significantly better functions than those produced by normal neural stem cells. Specifically, IFN-γ-hNSC-Exo can promote cell proliferation and survival and reduce cell apoptosis after ischemic stroke through specific exosomal miRNAs (hsa-miR-206, hsa-miR-133a-3p, and hsa-miR-3656) (Zhang et al., 2020). Another preclinical experiment confirmed that exosomes that transported NGF could reach the ischemic area to promote the recovery from ischemic stroke (Yang et al., 2020). The above studies can provide a theoretical basis for exosomes transporting cytokines to reach the SCI area to play a therapeutic role, but whether this idea can be realized is also restricted by how to safely and effectively deliver exosomes to the injured area. Therefore, in future scientific research, problems such as weak targeting ability and short half-life of exosomes need to be further studied.

#### Exosomes as Drug Delivery Vehicles for Traditional Chinese Medicines (TCMs)

In China, the use of TCMs has a history of thousands of years. In fighting against diseases for thousands of years, the Chinese people have gradually accumulated a wealth of medical knowledge through continuous practice and understanding. Because of the rapid advancement of science and technology, the research on TCMs continues to deepen. Previous preclinical and clinical findings have revealed that free TCMs can play an essential role in treating the pathophysiology of many diseases, including cancer (Wang R. et al., 2020), diabetes (Wang J. et al., 2020), Alzheimer’s disease (Zhang Z. et al., 2019), depression (Zhu et al., 2020), and COVID-19 (Pan et al., 2020; Tian J. et al., 2020). In addition, some researchers have begun to focus their study on how to treat CNS injuries more effectively by using exosomes as drug delivery vehicles, these carrying TCMs to specific areas and exerting therapeutic effects. For example, as early as 2016, a preclinical research study reported that a combined nano-formulation consisting of curcumin and embryonic stem cell exosomes (MESC-exo cur) could effectively reduce neurological damage, infarct volume, and edema in mice after ischemia reperfusion (IR) injury. This preclinical report demonstrated that the exosomes secreted by embryonic stem cells have the capability to transport the TCM component curcumin to the ischemic area and play a role in neurovascular recovery after ischemic injury (Kalani et al., 2016). In addition, after microglia were treated with resveratrol, resveratrol-primed exosomes (Exo + Res) were collected, and the exosomes were confirmed to improve the stability of resveratrol and reduce its degradation; most importantly, Exo + Res can stably pass through the BSCB and activate the PI3K/AKT signaling pathway. When the PI3K/AKT pathway was activated in the Exo + Res group, the survival rate and autophagy level of nerve cells in the SCI area were increased and the apoptosis level was decreased. It showed a more effective SCI alleviating effect than free resveratrol (Fan et al., 2020). The above evidence shows that the combination of exosomes and TCMs has the potential to treat SCI and has broad prospects in managing SCI. However, a significant amount of preclinical and clinical research is still needed.

### Surface-Functionalized Exosomes as Targeted Drug Delivery Vehicles for SCI

Exosomes may be used as endogenous drug delivery vectors to treat SCI because of their specialized characteristics, including low immunogenicity, inherent stability, high distribution efficiency, and capability of crossing the BSCB. However, to date, how to safely and effectively deliver exosomes specifically to the ischemic area remains the main obstacle to treating nervous system damage, including SCI, using exosomes. By limiting the transfer of the vast majority of neurotherapeutics and small molecules to the brain, the BBB and BSCB are the main obstacles to delivering macromolecular drugs to the nervous system. Because of the presence of the BBB and BSCB, when native exosomes were administered systemically to animals, they accumulated in the kidney, liver, and spleen, and were rapidly eliminated by renal filtration, bile excretion, and phagocytosis in the reticuloendothelial system (Barile and Vassalli, 2017). To improve the targeting efficiency, surface-functionalized exosomes that bear modified surface molecules are under development. In a preclinical analysis, researchers developed exosomes with a RVG peptide on the surface for neuron targeting, specifically Nerve Growth Factor (NGF) targeting to the ischemic cortex. The experimental results confirmed that the exosomes carrying NGF could maintain NGF with high stability, which can thus effectively function *in vivo* for a long time (Yang et al., 2020). In addition, in the field of glioma-targeted therapy, researchers use click chemistry technology to couple the exosome layer with neuropilin-1-targeted peptide (RGERPPR and RGE) to form an exosome with glioma-targeting function. The exosomes must provide a potential way to improve the management and evaluation of intracranial tumors (Jia et al., 2018). With the continuous deepening of research on exosomes with surface functionalization as drug delivery vehicles, surface-functionalized exosomes might provide a wide range of opportunities for their use in SCI treatment by their precise targeting.

### Hydrogel-Encapsulated Exosomes Have Great Potential in SCI Treatment

**FIGURE 4:**
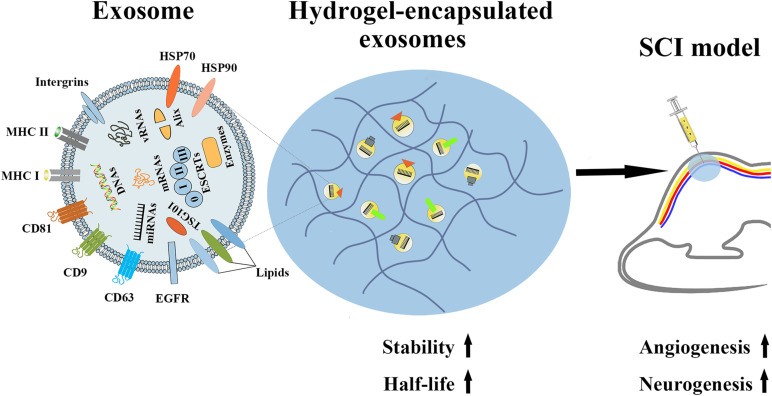
The properties of hydrogel-encapsulated exosomes and their potential functions in SCI. Hydrogel-encapsulated exosomes have the characteristics of higher stability and a longer half-life, and can promote angiogenesis and neurogenesis after SCI.

Although many studies have shown that exosomes can effectively alleviate tissue damage caused by ischemic stroke and SCI, whether *in vitro* or *in vivo*, ever more proof has shown that the therapeutic ability of systemic exosomes injection is primarily restricted by their short half-life and rapid clearance *in vivo*. According to a previous preclinical experiment, after exosomes secreted from B16BL6 melanoma cells were injected in mice, the exosomes were rapidly cleared by the mouse circulation, with a half-life of approximately 2 min (Takahashi et al., 2013). Therefore, researchers began to focus on slowing the release of exosomes *in vivo*, thereby increasing their *in vivo* half-life and enhancing their therapeutic potency. For example, the hydrogel UPy-hydrogel has ureidopyrimidinone (UPy) units coupled to poly (ethylene glycol) chains to function as a possible distribution network for exosomes. Following Upy-hydrogel administration to the physiological system, the continuous release of EVs from Upy-hydrogel was still detected 4 days later. Most importantly, the released EVs maintain their functional capacity. However, with the lack of the hydrogel, EVs are more likely to be affected by fat and skin tissue at the inoculation site. Therefore, we believe that the slow-release properties of hydrogels can provide EVs, including exosomes, with the property of slow-release and maintain their biological activity, and thus provide a broader prospect for the medical use of exosomes (Mol et al., 2019). In addition, in another preclinical experiment, the slow-release properties of hydrogel were used to experimentally test the slow release effects and the therapeutic impact of EV-loaded KMP2 hydrogel (KMP2-EVs) originated from MSCs *in vivo*. The results showed that hydrogel can effectively extend the half-life of exosomes *in vivo*, thereby enhancing the effects of exosomes on apoptosis, inflammation, and angiogenesis, thus showing the potential to improve renal function after I/R. This study emphasizes that hydrogel-encapsulated exosomes are promising cell-free therapies for tissue repair (Zhou et al., 2019). Additionally, hydrogel-encapsulated EVs, including exosomes, have displayed better effects than free exosomes in promoting cardiac repair (Chen et al., 2018; Han et al., 2019) and chronic diabetic wound healing (Wang et al., 2019) and promoting recovery from hindlimb ischemia (Zhang et al., 2018) and chronic liver failure (Mardpour et al., 2019). In the same way, we can attempt to apply hydrogel-encapsulated exosomes to alleviate the functional damage after SCI (Figure 4). We believe that such exosomes have tremendous promise in SCI treatment.

## Conclusion

Clinical data and experimental animal reports have shown that exosomes and exosomal miRNAs are closely associated with SCI. As bioactive substances, they have vital potential applications in disease diagnosis, prognosis, and treatment, and bring new hope for SCI treatment. However, at present, there are no unified standard research methods for exosomes and exosomal miRNAs nor accurate detection methods in disease processes. The results of *in vitro* studies and clinical trials that have been completed to date urgently need to be further verified *in vivo*. Therefore, we need to develop more advanced separation, extraction, and detection techniques of exosomes for greater in-depth research, and consider how to enhance exosome and exosomal miRNA sensitivity and specificity as biomarkers for various diseases. Exosomes as drug delivery vehicles will be a promising research and application direction, because exosomes can effectively cross the BBB, which could be utilized to treat and diagnose multiple CNS disorders, including SCI, and might become the next generation of miRNA-based therapy. How to improve the stability, targeting, and safety of exosomes will be a major topic in the research of therapeutic drugs.

## Author Contributions

LC conceived the structure of manuscript. JF, YZ, ZZ, and CG collected the data. JF and YZ wrote the manuscript. AW revised the manuscript. All authors contributed to the article and approved the submitted version.

## Conflict of Interest

The authors declare that the research was conducted in the absence of any commercial or financial relationships that could be construed as a potential conflict of interest.
